# Transparent TiO_2_/Cu/TiO_2_ Multilayer for Electrothermal Application

**DOI:** 10.3390/ma14041024

**Published:** 2021-02-22

**Authors:** Jingjing Peng, Changshan Hao, Hongyan Liu, Yue Yan

**Affiliations:** Beijing Engineering Research Center of Advanced Structural Transparencies for the Modern Traffic System, Beijing Institute of Aeronautical Materials, Beijing 100095, China; dashan184504@163.com (C.H.); homyeeliu@126.com (H.L.); yueyan@biam.ac.cn (Y.Y.)

**Keywords:** transparent conductive oxides, optical property, electrical property, annealing, electrothermal heating

## Abstract

Highly transparent indium-free multilayers of TiO_2_/Cu/TiO_2_ were obtained by means of annealing. The effects of Cu thickness and annealing temperature on the electrical and optical properties were investigated. The critical thickness of Cu mid-layer with optimal electrical and optical properties was 10 nm, with the figure of merit reaching as high as 5 × 10^−3^ Ω^−1^. Partial crystallization of the TiO_2_ layer enhanced the electrical and optical properties upon annealing. Electrothermal experiments showed that temperatures of more than 100 °C can be reached at a heating rate of 2 °C/s without any damage to the multilayers. The experimental results indicate that reliable transparent TiO_2_/Cu/TiO_2_ multilayers can be used for electrothermal application.

## 1. Introduction

Transparent conductive oxides (TCOs) with a unique combination of high electrical conductivity and visible transparency are indispensable materials in various areas [1,2,3,4,5,6]. The most traditional material used in these areas is indium tin oxide (ITO), which has a low resistivity (about 10^−4^ Ω·cm) and high transmittance (about 80%) [7,8]. However, the finite supply of indium and its poor compatibility with flexible substrates seriously restrict its application. Promising alternative indium-free materials, including pure SnO_2_, ZnO, or ZnO doped with metals, Nb_2_O_5_, and TiO_2_, still need improvement and are far from extensive application.

The oxide/metal/oxide (OMO) multilayer has emerged as a competitive structures alternative to single layers [1,9,10,11,12]. The OMO multilayer has a low sheet resistance and high transparency that are comparable to that of ITO. The thin metal layer in the middle reduces the resistivity of the multilayer, which should be uniform, thin, and continuous to reduce loss in optical transmittance. Ag, which has optimal conductivity and excellent optical transmittance, had been widely investigated [9,10]. Earth-abundant Cu with comparable properties but lower price has been considered as a competitive candidate for Ag to reduce cost. In the aspect of oxidation resistance, Cu layer performs better than Ag [12,13,14,15]. Thus, Cu can be used as a middle layer in OMO structure in devices exposed to humid and corrosive environment.

In the OMO structure, oxide layer is critical given that it is employed to enhance transmittance, reduce environmental oxidation of thin metal film, and promote continuous growth of thin metal films. Titanium dioxide, possessing strong chemical bond and mechanical stability, can be an excellent protective layer over the metal layer in dielectric/metal/dielectric design in electrothermal application. High optical transmittance, high refractive index, and wide band gap make TiO_2_ ideal for improving the transmittance of OMO structure [1,14].

In spite of its promising properties, limited research investigated the optimization of TiO_2_/Cu/TiO_2_ multilayers. Adequate efforts have not been exerted to address how external stimuli affect their properties. In phase changing materials, properties can be switched by external stimuli, as amorphous and crystalline phases display differences both in optical and electrical properties [16,17,18,19]. Similar technologies can be applied in OMO structures as amorphous crystallized oxide layers exhibit different optical properties [20]. The crystallization of the oxide layer will cause a difference in the electron scattering in the metal layer, which result in different electrical property. Annealing is a feasible method to render crystallization in ultrathin films, thus the property of OMO structures is promising to be greatly enhanced by means of annealing.

Apart from the widespread application of TCO in solar cell and panel display, OMO structure hold the potential for application in defogging and anti-icing of the window, aircraft, and windshield [21,22,23,24,25]. Electrothermal films transform electric energy to heat to reduce ice or frog at low temperature. Besides, electrothermal films have to be highly transparent. Carbon nanotubes and grapheme are widely investigated for such an application [26,27,28]. Despite their good properties, the problem of uncontrolled synthesis and high cost of macroscopic assemblies remains to be solved [29]. OMO structures can be conveniently produced at a large scale at low cost, so OMO structures are promising candidates for electrothermal film. Additionally, OMO structures possess the same outstanding thermal, electrical, and optical properties as well. The electrothermal response speed, operating voltage, temperature distribution, and thermal stability of OMO as electrothermal materials are critical issues to be explored. In this paper, we investigated the magnetron-sputtered TiO_2_/Cu/TiO_2_ multilayers on glass substrates for electrothermal application. The thin film of TiO_2_ and Cu was deposited on glass substrates at room temperature utilizing stoichiometric TiO_2_ and Cu targets, respectively. The effect of annealing of TiO_2_/Cu/TiO_2_ multilayer was investigated in detail. By tuning the multilayer thickness and annealing, a Cu-based OMO structure with excellent electrothermal property was developed.

## 2. Materials and Methods

The TiO_2_/Cu/TiO_2_ multilayer was deposited by magnetron sputtering (JCP500, Beijing Technol Science Co.,Ltd., Beijing, China) at room temperature on glass substrates. Before sputtering, all substrates were ultrasonically cleaned in alcohol and deionized water for 5 min separately and then dried with nitrogen gas. Pure Cu and stoichiometric TiO_2_ targets were used to sputter the TiO_2_/Cu/TiO_2_ multilayer. The magnetron sputtering chamber was pumped down to 10^−5^ Pa with a turbo molecular pump. The flow of gases was controlled with mass flow controllers. Prior to TiO_2_ deposition, the target was pre-sputtered under the deposition conditions for 10 min, during which the substrates were covered by a moveable shutter. The underlying TiO_2_ layer was grown by a sputtering power of 20 W at a pressure of 0.35 Pa, with Ar and O_2_ gas flow of 4 and 2 sccm, respectively. The chamber was pumped for 10 min to avoid the oxidation of Cu layer by remnant O_2_ gas after the growth of the TiO_2_ layer. Then, the Cu target was pre-sputtered for 10 min, and the Cu middle layer was then grown by a sputtering power of 15 W at a pressure of 0.35 Pa, with Ar gas flow of 6 sccm. The overlying TiO_2_ was deposited under the same parameters as the underlying TiO_2_ layer without presputtering. During the entire deposition process, the substrate was not intentionally heated. An adhesive tape was fixed on the substrate before sputtering and then removed after sputtering to form a step, the height of which corresponds to the thickness of the film. The thickness of each film layers were then examined by Profilometer (P7, KLA Tencor, San Francisco, CA, USA), which gave accurate measurement of thickness with uncertainty less than 2 nm. After film growth, the TiO_2_/Cu/TiO_2_ multilayers were annealed at different temperatures (300 °C and 400 °C) in nitrogen ambient atmosphere for 30 min. Surface roughness was detected by atomic force microscopy (AFM; Bruker Co. Edge, Karlsruhe, Germany) in the tapping mode with a scanning area of 5 μm × 5 μm. Root-mean-squared roughness value was calculated over the scanned area. The transmittance spectra were measured with an ultraviolet-visible-near infrared spectrophotometer (VARIAN 5000, Lincolnshire, IL, USA) over a wavelength range of 400–2000 nm at an interval of 1 nm. By calculating the mean arithmetical value, the average transmittance of multilayers at visible wavelengths (from 400 to 700 nm) was obtained. Electrical property was measured using Ven der Pauw method using commercial equipment (HL5500, Nano Metrics, Milpitas, CA, USA). Film crystal structure was investigated using a grazing incidence X-ray mode diffractometer (AU5, Rigaku, Tokyo, Japan). During measurement, the incident angle was fixed at 5° with respect to the substrate surface to minimize interference from substrates. An infrared thermography (TIM QVGA, Micro-epsilon, Messtechnik GmbH&Co.KG, Haunetal, Germany) was used to monitoring temperature with a precision of ±2%.

## 3. Results

**Figure 1:**
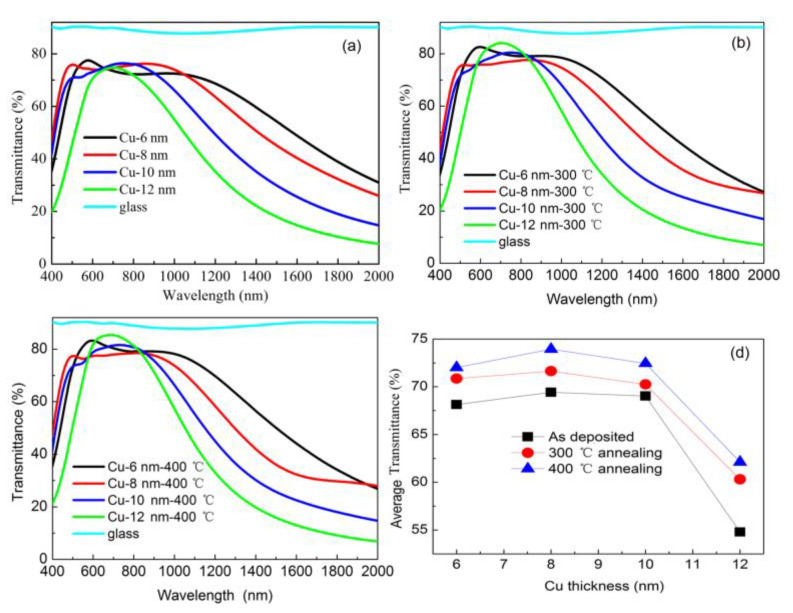
Transmittance spectra of (**a**) as-deposited multilayers and multilayers (**b**) annealed at 300 °C and (**c**) 400 °C. (**d**) Average transmittance of multilayers as a function of Cu thickness.

For the TiO_2_/Cu/TiO_2_ multilayer, the thickness of TiO_2_ was kept at 30 nm, whereas that of Cu varied from 6 to 12 nm. Figure 1 shows the transmittance spectra of multilayers with different Cu thicknesses and annealing at different temperatures. In the figure, the spectra varied a lot with different Cu thickness as thicker Cu layer showed narrower bandwidth. Our results highlighted the domination of Cu interband transition, in agreement with Cu nanocube systems. [30] As shown in Figure 1a, the bandwidth of transmittance at visible wavelengths was narrowed by increasing the thickness of the Cu layer from 6 to 12 nm and was mainly caused by the strong free carrier absorption of the thick Cu layer. For wavelengths above 700 nm, transmission descended with the increase in Cu thickness due to the increased bound electrons available for excitation with the thick Cu film. The transmission of all as-deposited multilayers showed no rise above 80%. High-temperature annealing was widely used and a feasible method to increase transmission and conductivity. As suggested by other literature [1,31], high temperatures above 500 °C induced partial oxidation of the Cu layer, which was fatal for transmission and conductivity. The annealing temperature had to be a compromise between an increase in transmission and partial Cu oxidation. To avoid the partial oxidation of Cu thin film at high temperatures, temperatures of 300 °C and 400 °C were employed in our experiments, with further consideration that a temperature lower than 300 °C poorly enhanced transmission. The multilayers were annealed in nitrogen ambient atmosphere to reduce oxidation. Figure 1b,c displayed the transmittance spectra of annealed multilayers. Although the shape of transmittance spectra showed no considerable change, the peak of every curve elevated to a higher value, with several surpassing 80%. By calculating mean arithmetical value, the average transmittance of multilayers at visible wavelengths (from 400 to 700 nm) was shown in Figure 1d and summarized in Table 1. The transmittance of the as-deposited multilayers was elevated to a higher level by annealing. For the as-deposited multilayers with 12 nm Cu, the average transmittance was around 55%, which can be enhanced to 63.11% by 400 °C annealing. This finding indicated that the heavy reflection from the Cu metal layer could be reduced by high-temperature annealing. For the multilayers with a thin Cu layer, the as-deposited sample exhibited substantially high transmittance due to decreased reflection from the Cu layer. In addition, transmittance can be further increased to a higher value by annealing. All these results indicated that annealing reduced the reflection from Cu metal layer regardless of thickness. Furthermore, the average transmittance of 74.23% was observed in multilayers with 8 nm Cu and annealed at 400 °C; this condition is almost comparable to that of glass without coating. The average transmittance of annealed multilayer at 400 °C was enhanced by 5% compared with the as-deposited one.

**Figure 2:**
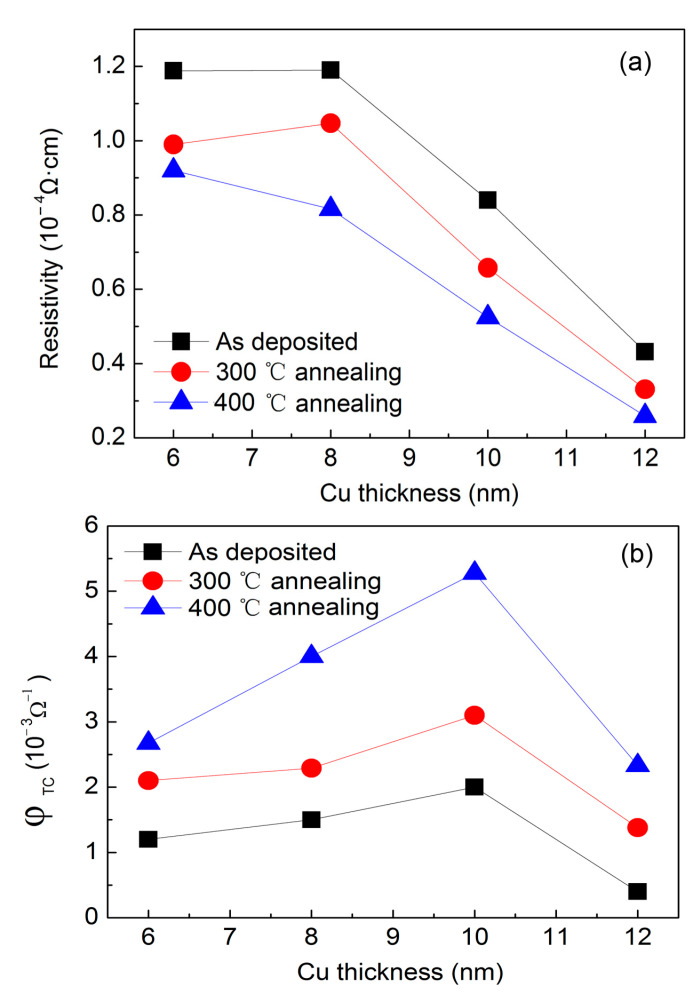
(**a**) Resistivity of multilayers and (**b**) figure of merit (FOM) as a function of Cu thickness.

Figure 2a exhibited the resistivity of the multilayer as a function of Cu thickness. For the as-deposited multilayers with a Cu thickness lower than 8 nm, the resistivity remained as high as 1.2 × 10^−4^ Ω·cm due to grain-boundary scattering of electrons between small Cu islands. With the increase in Cu thickness to 12 nm, resistivity dropped dramatically to 0.4 × 10^−4^ Ω·cm, which implies the formation of a contiguous Cu layer. After annealing, the resistivity can be further reduced compared with that observed in 300 °C annealed multilayers, whereas a large reduction in resistivity was observed in 400 °C annealed multilayers. For samples with 12 nm Cu annealed at 400 °C, resistivity was as low as 0.25 × 10^−4^ Ω·cm. Further reduction in resistivity might have occurred if the annealing temperature was further elevated to above 400 °C. However, a temperature higher than 400 °C was not suggested because partial oxidation of Cu metal thin film at high temperature wouldcause damage to transparency [31].

To gain further insights into the effect of annealing, we calculated the figure of merit (FOM) in accordance with the relationship (Equation (1)) defined by Haacke [9,32]:

(1) $$\psi_{\mathrm{TC}}=\frac{T_{\mathrm{av}}{}^{10}}{R_{\mathrm{sh}}}$$

where *T*_av_ is the average transmittance in the visible-light range and *R*_sh_ is the sheet resistance. *R*_sh_ is calculated by *R*_sh_ = ρ × *t*, whereas ρ and *t* are the resistivity and thickness of the Cu layer, respectively, in the condition that only the Cu layer contributes to conductivity.

Figure 2b displayed the FOM as a function of Cu thickness. The FOM remained low for the as-deposited multilayer owing to the low transmittance and high sheet resistance. Through annealing, transmittance was enhanced with reduced sheet resistance. The figure also showed that the FOM was boosted by annealing. A maximum FOM of as high as 5 × 10^−3^ Ω^−1^ was observed in the multilayer with 10 nm Cu layer annealed under 400 °C.

**Figure 3:**
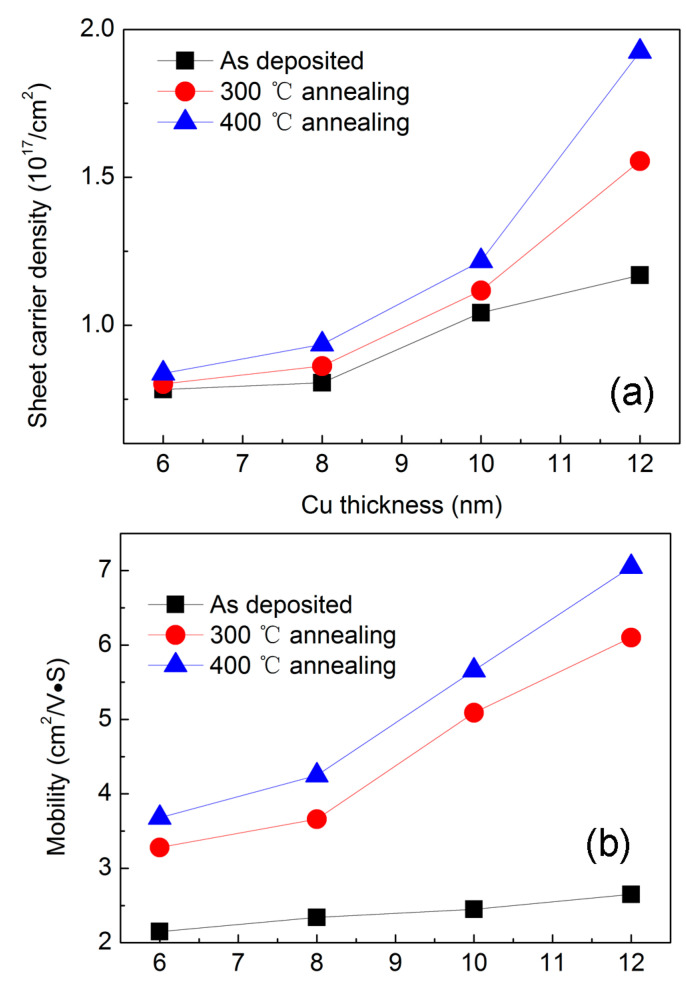
(**a**) Sheet carrier density and (**b**) mobility as a function of Cu thickness.

The above experimental results gave firm evidence that annealing not only reduced resistivity but also elevated transmittance. Sheet carrier density and mobility were measured by the Vender Pauw method, as shown in Figure 3a,b. When Cu thickness increased from 6 to 12 nm, a moderate increase was recorded in sheet carrier density from 0.78 × 10^17^ to 1.16 × 10^17^ cm^−2^, whereas the mobility remained almost stable around 2.5 cm^2^/V·S. Upon annealing, the mobility was greatly enhanced to a higher value. When we fitted the curve, there was no exponential dependency between the mobility and Cu thickness. As for the sheet carrier density, there was no substantial change between as-deposited and annealed multilayers with 8 and 10 nm Cu layers. Figure 2a showed that resistivity decreased upon annealing. All these findings hinted that reduced resistivity was mainly caused by high mobility. As indicated by Dhar et al. [10], mobility can be affected by phonon, interface, grain-boundary, surface, and ionized impurity scatterings. The Cu layer grown on an amorphous TiO_2_ layer should follow the island growth mechanism of the Volmer–Weber model. If the TiO_2_ layer becomes crystallized upon annealing, the thin Cu layer became smooth and showed reduced scattering and enhanced mobility. Thus, a reduced resistivity was expected. In the aspect of transmittance, a smooth interface helped to reduce light scattering and reflection from the interface and promoted the increase in transmittance.

**Figure 4:**
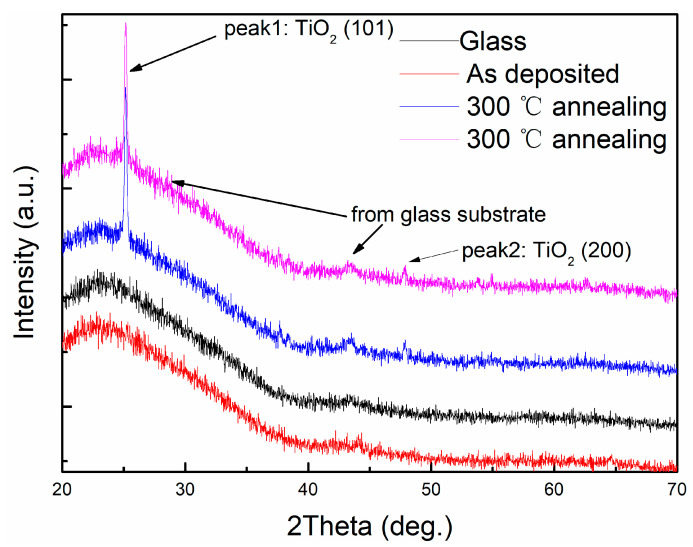
XRD patterns of multilayers annealed at different temperatures.

**Figure 5:**
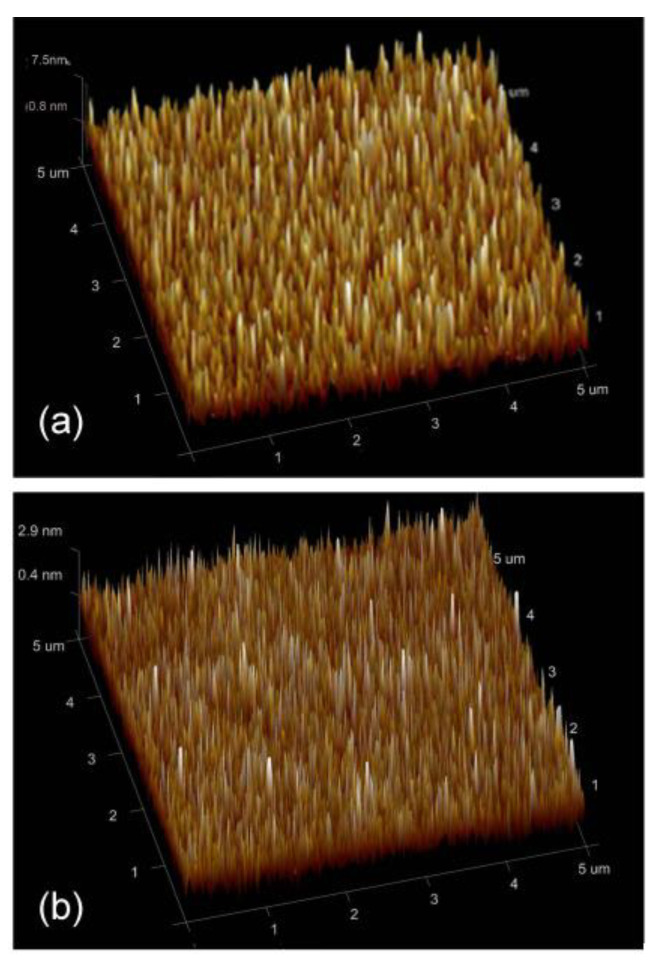
Atomic force microscopy (AFM) image of (**a**) as-deposited multilayer and that (**b**) annealed at 400 °C.

To check whether the TiO_2_ layer becomes crystalized upon annealing, we performed grazing incidence X-ray diffraction (XRD) (Figure 4). To exclude the effect from the glass substrate, we also performed XRD of the substrates. One high broad peak and one small peak originated from the substrate. The as-deposited multilayer displayed the same peak as the substrate, indicating the amorphous nature of the TiO_2_ and Cu layer. The annealed multilayer contained a distinct peak at 25.16°, which accounted for the anatase TiO_2_ (101) peak. Another small but visible peak at 48° corresponded to the anatase TiO_2_ (200) peak. The XRD results proved that the TiO_2_ layer might be crystallized to a certain extent. AFM was conducted on the as-deposited and annealed samples to observe how the surface of the layer changed with the crystallization of the TiO_2_ layer. Three-dimensional AFM images with the Z-axis highlighting the height were shown in Figure 5a,b. In an effort to elucidate the difference in surface roughness of films before and after annealing, root-mean-squared roughness value was calculated over the scanned area. Figure 5a showed an uneven surface with a roughness of 0.67 nm. Figure 5b presented a notably smooth surface with a roughness of 0.45 nm. Combined with the XRD results, it was concluded that the TiO_2_ layer was crystallized and became smoother upon annealing, thus promoting the smooth surface in the Cu layer. As mentioned above, reduced scattering from the interface led to high conductivity and transmittance.

**Figure 6:**
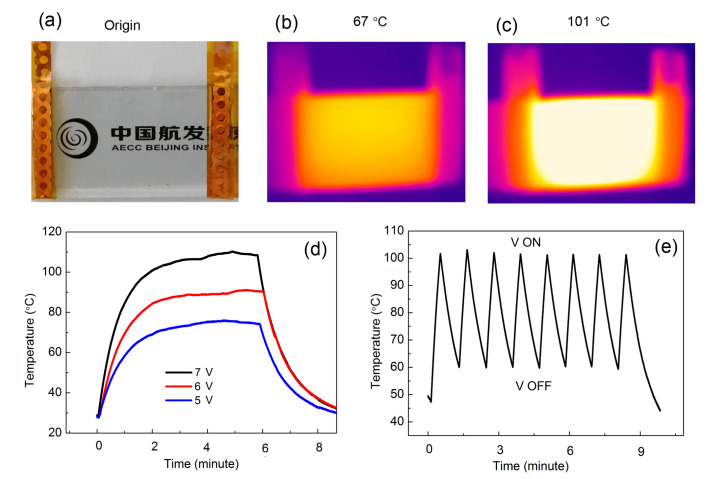
(**a**) Image of electrothermal sample. Electrothermal samples at (**b**) 67 °C and (**c**) 101 °C. (**d**) Sample temperature as a function of time under electrical heat and (**e**) temperature cycles with voltage ON and OFF.

Thus far, we proved that the TiO_2_/Cu/TiO_2_ multilayer boosted transmittance and increased electrical conductivity simultaneously. Next, we tested the electrothermal performance, which was important to avoid frosting and freezing of airplane glass surface under extreme weather conditions. Figure 6a showed the sample for electrothermalexperiment. The TiO_2_/Cu/TiO_2_ multilayer with 10 nm Cu was selected because of its good transparency and conductivity. On the two edges of the sample, an Au electrode was deposited to improve the conductivity to the Cu electric wire, on which the electric voltage was directly applied. Figure 6b,c displayed the infrared image of samples with operating voltages. The figures show that temperature was evenly distributed, with the value reaching as high as 101 °C in the sample surface without any over-heated spot or area. Therefore, resistivity was even in the sample area. This finding was important for applications given that over-heated spots or areas cause device failure. The smoother the temperature during electrothermal process, the better the performance in avoiding frosting and freezing of airplane glass. We did not apply temperatures higher than 101 °C, because 100 °C is sufficient to prevent frosting and freezing. To evaluate the performance of the multilayer, we monitored one spot on the sample to observe how its temperature responded to the applied voltage. As the temperature was evenly distributed on the sample, the monitored spot can be selected randomly. Figure 6d shows the results. Upon applying 5 V on the sample, the temperature of the spot rose quickly to 73 °C within 110 S, which is similar to the previously reported graphene’s response time of about 60–150 s [26,27]. When the voltage was kept stable, the temperature was also stable with a fluctuation of less than 3 °C. Similarly, when applying 6 and 7 V voltage on the sample, the temperature of the spot rose quickly to 88 °C and 107 °C, respectively. The temperature remained stable afterward. The steady temperature (*T*_steady_) increased from 73 to 107 °C while increasing the operating voltage from 5 to 7 V. The response time, defined as the time to reach approximately 90% of the steady temperature from room temperature, is one of the key factors for electrothermal performance. The higher the voltage, the higher the steady temperature and the faster the response speed. Benefiting from the low resistance of the films, the potential of decreasing response time just by applying a larger operating voltage can be further explored. The experiment firmly proved that temperature could be controlled by direct voltage, which is convenient in practical applications.

Figure 6e displayed the quick response of temperature to voltage. Upon applying 10 V onto the sample, the temperature ascended steeply to 100 °C. A maximum heating rate as large as 2 °C/s can be reached, which is even superior to heating rate of carbon nanotube electrothermal systems [27,28,29]. After reaching 100 °C, we turned the voltage OFF, and the temperature descended quickly at a cooling rate of 0.77 °C/s. We performed the experiment at 10 V in eight cycles, which all showed similar characteristics. As we further did the same experiments up to 200 cycles, similar results were obtained without cracking of the film or failure of device. The results were reproducible on several other samples. The results proved that our sample remained highly stable under frequent operations with duration larger than 200 cycles.

## 4. Conclusions

In conclusion, highly transparent and conductive TiO_2_/Cu/TiO_2_ multilayers were obtained by annealing at 400 °C. The smoother TiO_2_/Cu interface caused by partial crystallization of TiO_2_ upon annealing not only reduced electron scattering and enhanced mobility, but also reduced light scattering and reflection from the interface. Annealed TiO_2_/Cu/TiO_2_ multilayers exhibited a figure of merit as high as 5 × 10^−3^ Ω^−1^. Devices made of TiO_2_/Cu/TiO_2_ multilayers displayed optimal electrothermal properties in respect of a fast electrothermal response, small operating voltage and even temperature distribution. Electrothermal experiments showed that device temperatures of more than 100 °C at a heating rate of 2 °C/s with 10 V operating voltage can be reached without any damage to the multilayers. Successive supply cycles with voltages ON and OFF demonstrated the repeatability and reliability of the device. This work gave firm evidence that TiO_2_/Cu/TiO_2_ multilayers can be used as reliable transparent material for electrothermal applications.

## Figures and Tables

**Table 1:** Summary of average transmittance of multilayers at visible wavelengths (from 400 to 700 nm).

Cu Thickness	As Deposited	300 °C Annealing	400 °C Annealing
6 nm Cu	68.13%	70.86%	72.01%
8 nm Cu	69.42%	71.64%	74.23%
10 nm Cu	69.03%	70.05%	72.45%
12 nm Cu	54.89%	60.31%	63.11%

## Data Availability

The data presented in this study are available on request from the corresponding author.
